# A dynamically tunable terahertz human serum albumin biosensor based on Dirac semimetal nanozyme

**DOI:** 10.3389/fbioe.2025.1736291

**Published:** 2026-01-09

**Authors:** Ling Chen, Qiaohong Yao, Jie Chen, Yuxiang Peng, Jiao Xu, Qiang Fu

**Affiliations:** 1 The Central Hospital of Xiangtan, The Amiliated Hospital of Hunan University, Xiangtan, China; 2 Institute of Mathematics and Physics, and Hunan Province Key Laboratory of Materials Surface & Interface Science and Technology, Central South University of Forestry and Technology, Changsha, China; 3 School of Information Science and Engineering, Hunan Women’s University, Changsha, China

**Keywords:** optical biosensor, human serum albumin, dirac semimetal, terahertz, herestucture

## Abstract

This study presents a tunable terahertz biosensor based on a hybrid architecture integrating bulk Dirac semimetal (BDS) with a photonic crystal for the label-free and highly sensitive detection of human serum albumin (HSA). The sensor exhibits a sharp Fano resonance resulting from the synergistic coupling between a defect mode and the BDS-mediated plasmonic response, with its resonance frequency being highly sensitive to variations in the refractive index of the sensing layer. Electric field simulations confirm significant field confinement and enhancement within the sensing region. The electrically tunable property of BDS allows dynamic reconfiguration of the sensor’s sensitivity by adjusting the Fermi level (0.1–0.4 eV), though at the expense of a reduced figure of merit (FOM) due to broader resonance peaks and increased loss, necessitating a balance in practical implementations. Structural parameter analysis reveals that sensitivity is inversely proportional to the sensing layer thickness and gradually decreases with increasing refractive index. The proposed biosensor achieves a reflection dip deeper than 99% near 1 THz, with an angular sensitivity of 247.5°/RIU for minute refractive index changes (Δn = 0.002), demonstrating high sensitivity and excellent electrical tunability. This platform demonstrates pioneering potential in integrating diagnostic-treatment nanotechnology. Due to the powerful local field enhancement generated by the BDS photonic crystal heterostructure, the imaging sensitivity of the contrast agent based on nanoenzymes has been significantly improved. Moreover, the profound local field enhancement and dynamic tunability of this platform suggest its potential for real-time monitoring and regulation of the catalytic efficiency of nanoenzymes, which could address a fundamental challenge in therapeutic applications. Combined with its inherent biocompatibility and strong detection capabilities, this framework proposes a viable pathway toward the clinical translation of nanoenzyme technology. Our work thus establishes a foundational platform that paves the way for future multifunctional theranostic systems capable of combining sensitive biomarker monitoring with enhanced therapeutic effects.

## 1 Introduction

Optical biosensing technology has emerged as a key platform for biomarker detection, owing to its high sensitivity, rapid response, and label-free operation (Zhang et al., 2024; Lee et al., 2025). By converting subtle biological responses or weak biomolecular interactions into measurable optical parameters, this technology not only offers significant advantages including non-invasive operation, label-free detection, and non-destructive analysis but also demonstrates excellent signal stability and detection specificity (Altug et al., 2022). These merits have established optical biosensing as a critical enabler for screening disease-related biomarkers in clinical diagnosis and treatment (Kaur et al., 2022). It facilitates rapid, sensitive, and often real-time detection of disease markers, pathogenic factors, and therapeutic drug levels in settings such as emergency departments, outpatient clinics, or laboratories (Rubin et al., 2018; Rawson et al., 2021). Meanwhile, Human serum albumin (HSA), the most abundant protein in plasma, plays pivotal physiological roles in maintaining colloidal osmotic pressure, transporting nutrients and metabolites, and regulating homeostasis (Duan et al., 2025; Płuciennik et al., 2025; Linero-Artiaga et al., 2025). Clinical evidence demonstrates a strong correlation between abnormal HSA levels and various pathological conditions, making its quantitative detection crucial for diagnosing liver diseases such as cirrhosis (Jagdish et al., 2021) and renal disorders like nephrotic syndrome (Ponticelli and Moroni, 2023), as well as for prognostic assessment and screening for malnutrition (Deng et al., 2025; Yang et al., 2025).

Recent breakthroughs in micro-nano fabrication have significantly advanced the miniaturization and integration of sensor architectures, giving rise to various high-performance sensing structures including photonic crystals (Xavier et al., 2018), carbon nanotube arrays (Yuan et al., 2019), and terahertz plasmons (Xing et al., 2021). Among these sensing mechanisms, Fano resonance has garnered considerable attention due to its characteristic asymmetric line shape and exceptional environmental sensitivity (Rawat et al., 2025; Ni et al., 2025). This physical phenomenon arises from the coherent interference between discrete and continuum states, generating resonance peaks with steep slopes that are highly sensitive to minute changes in the surrounding refractive index (Zhao et al., 2025). Compared to traditional Lorentzian resonances, Fano resonance exhibits notable advantages in biosensing applications, including superior sensitivity, enhanced signal-to-noise ratio, and improved robustness against interference (Limonov et al., 2017; ElKabbash et al., 2021). It has been successfully deployed in diverse applications such as protein detection (Barth and Lee, 2025), nucleic acid analysis (Salama et al., 2024), and cellular activity monitoring (Wang et al., 2024).

Concurrently, novel quantum materials present new opportunities for further enhancing biosensor performance. Dirac semimetals, particularly bulk Dirac semimetals (BDS), have attracted significant interest due to their unique electronic band structure and tunable dielectric properties, which enable strong localized field enhancement and electrically controllable optical responses in the terahertz regime (Cuozzo et al., 2024; Cao et al., 2025; Sahu et al., 2023). These characteristics make BDS an ideal platform for constructing high-performance plasmonic sensors. Research indicates that BDS-based sensors significantly outperform conventional gold-based surface plasmon resonance sensors in detection sensitivity (Yu et al., 2025; Xie et al., 2025). More importantly, the Fermi level of BDS can be modulated through external electric fields or chemical doping, enabling dynamic reconfiguration of sensor performance and environmentally adaptive detection (Anh et al., 2024).

The integration of Fano resonance-based sensing mechanisms with novel Dirac semimetal materials represents a promising direction for developing next-generation high-sensitivity biosensors. Through careful design of the sensor architecture, high-quality-factor Fano resonances can be excited, enabling high-precision analysis of biomolecular binding events. In this study, we propose a terahertz biosensor based on a BDS-photonic crystal hybrid structure for label-free detection of HSA. The sensor leverages synergistic coupling between the photonic crystal defect mode and the BDS-supported plasmonic mode to generate a pronounced Fano resonance effect, achieving strong field confinement and signal amplification within the sensing region. We systematically investigated the influence of the BDS Fermi level on sensor performance and optimized key parameters including sensing layer thickness and refractive index, achieving an optimal balance between sensitivity and figure of merit. The developed sensor exhibits high sensitivity, electrical tunability, and excellent stability, offering a promising platform for clinical monitoring of hepatic and renal function-related diseases.

## 2 Methods

**FIGURE 1:**
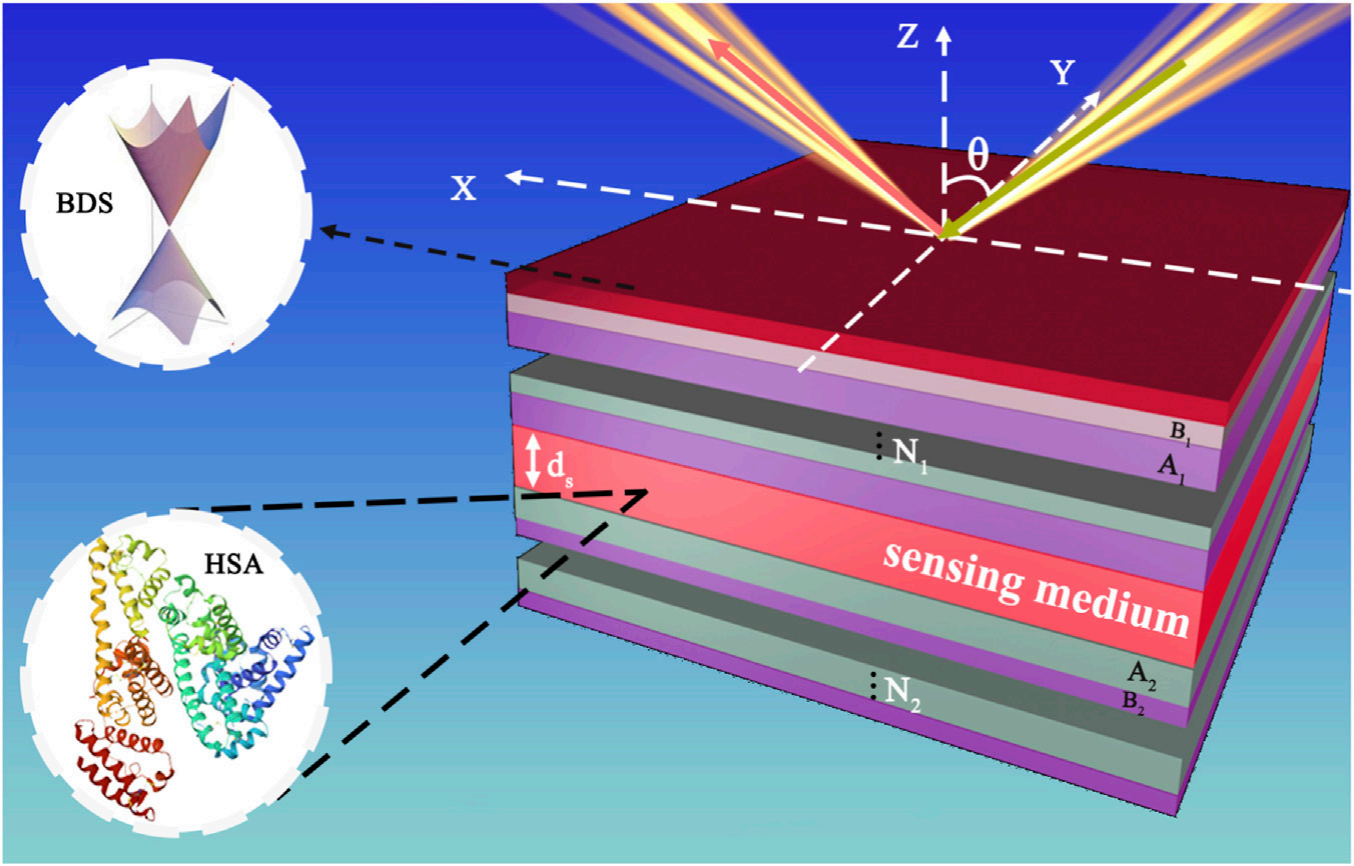
Schematic diagram of a human serum albumin biosensor based on heterostructure with BDS.

A heterostructure is proposed herein, comprising Bulk Dirac Semimetal (BDS), photonic crystal 1 (PhC1), a sensing layer, and photonic crystal 2 (PhC2), as depicted in Figure 1. The BDS is positioned at the top of the structure, and is the first component encountered by incident light after it traverses the air. To enhance the practical applicability of the biosensor model, inlet and outlet channels for the sensing liquid are integrated within the sensing medium layer. PhC1 and PhC2 are fabricated using alternating layers of real optical materials A (TiO_2_) and B (Si). These photonic crystals comprise two distinct dielectrics, A and B, with their refractive indices and thicknesses denoted $n_{a},d_{a},n_{b},d_{b}$ accordingly. The refractive indices of dielectrics A and B are assumed to be non-dispersive, set as: $n_{a}=2.35$ and $n_{b}=1.46$ ; their thicknesses are set to $d_{a}=31.1\;\mu m$ and $d_{b}=42.5\;\mu m$ , respectively. The sensing layer is sandwiched between the two photonic crystals, with the photonic crystal period set to “ $N_{1}=N_{2}=5$ ”. Human serum albumin (HSA) is chosen as the sensing layer owing to its excellent biocompatibility, abundant ligand-binding sites, and stable physicochemical properties. It has a refractive index of $n_{s}=1.365\pm 0.001$ and a thickness of $d_{s}=487\;\mu m$ (Malmsten, 1994), represents the effective refractive index of a hydrated protein film in the THz frequency range. Its use inherently accounts for the significant hydration shell and the specific dielectric dispersion of the protein-water complex at our operational frequency of 1 THz, thereby ensuring the physical relevance of our model.

The proposed heterostructure can be realized by first growing high-quality cadmium arsenide (Cd_3_As_2_)films via molecular-beam epitaxy to serve as the Dirac semimetal platform (Rice et al., 2022). Subsequently, a photonic crystal structure composed of alternating TiO2and Si layers can be fabricated on the BDS film through a combination of electron-beam lithography for precise patterning and layer-by-layer deposition for structural integrity (Kim and Kuljanishvili, 2025; Yi, 2025). Finally, microfluidic channels would be integrated for controlled liquid sample delivery to the sensing layer, thereby ensuring a practical and integrated device that leverages recent advances in heterostructure fabrication (Safarkhani et al., 2024).

Considering the 3D nature of the BDS, bulk conductivity is used to characterize it (Ooi et al., 2019). In particular, we chose 1 THz frequency range, where the intraband contribution is dominant for the doped material, as interband transitions are suppressed at these low frequencies relative to the Fermi level. The linear intraband optical conductivity of the BDS can be analytically derived from the semiclassical Boltzmann transport equation under the relaxation time ap-proximation, given by:

$$\sigma =\sigma_{0}\frac{4}{3\pi^{2}}\frac{\tau }{1-i\omega \tau }\frac{{\left(k_{B}T\right)}^{2}}{\hbar^{2}v_{F}}\left[2Li_{2}\left(-e^{-\frac{E_{F}}{k_{B}T}}\right)+{\left(\frac{E_{F}}{k_{B}T}\right)}^{2}+\frac{\pi^{2}}{3}\right],$$ (1)

where $\sigma_{0}\equiv \frac{e^{2}}{4\hbar }$ and $Li_{s}\left(z\right)$ is a multiple logarithmic function. ω is the angular frequency of the incident beam, τ represents the relaxation time, T is the temperature, $k_{B}$ and ℏ are Boltzmann constant and reduced Planck constant, respectively, $v_{F}$ is the Fermi velocity of the electron, and $E_{F}$ is the Fermi energy. From the above expression, it is not difficult to find that the conductivity of BDS can be dynamically adjusted by the Fermi energy $E_{F}$ .This expression indicates that the conductivity of the BDS can be dynamically tuned by adjusting the Fermi energy, providing a means to develop dynamically tunable biosensors. Similar to the dynamic regulation scheme for graphene, an external voltage can be applied to modify the Fermi energy of the BDS and thus regulate its conductivity. In practice, this adjustment can be realized by incorporating electrodes between the BDS and the substrate. For subsequent calculations, the initial parameters of the BDS are set as $E_{F}=0.01\;eV$ and τ=1 ps .

To evaluate the reflection characteristics of the structure and thereby determine its sensing performance, the conventional and reliable transfer matrix method is utilized. For simplicity, the analysis is focused on the TM polarization mode. The electromagnetic boundary condition at the BDS-dielectric interface is treated using a transfer matrix formalism applicable to conductive interfaces characterized by a surface conductivity. This approach, commonly used for graphene, is directly applicable here as the BDS layer supports similar surface-confined modes in the terahertz regime (Federici and Moeller, 2013):

$$D_{i\to A}=\frac{1}{2}\left[\begin{array}{c} 1+\eta_{\text{iA}},\\ 1‐\eta_{\text{iA}}, \end{array}\begin{array}{c} 1‐\eta_{\text{iA}}\\ 1+\eta_{\text{iA}} \end{array}\right],$$ (2)

where $\eta_{\text{iA}}=\frac{\;\varepsilon_{i}k_{\text{Az}}}{\varepsilon_{A}k_{\text{iz}}}$ , $k_{\text{iz}}$ and $k_{\text{Az}}$ are the wave vector components of electromagnetic waves propagating in the air layer and dielectric A, respectively. The transfer matrix method was implemented with the interface matrix D (Equation 2) and the propagation matrix P_j_ for a layer j, given by:

$$p_{j}=\left[\begin{array}{cc} e^{-{ik}_{jz}d_{j}} & 0\\ 0 & e^{{ik}_{jz}d_{j}} \end{array}\right],$$ (3)

where k_jz_ is the wave vector component normal to the layers and is the layer thickness. By combining the propagation matrices of electromagnetic waves in each dielectric layer, the transmission matrix of the heterostructure is obtained as:

$$M=D_{i\to BDS}p_{BDS}D_{BDS\to A}{\left(p_{A}D_{A\to B}p_{B}D_{B\to A}\right)}^{4}p_{A}D_{A\to B}p_{B}D_{S\to B}{\left(p_{B}D_{B\to A}p_{A}D_{A\to B}\right)}^{4}p_{B}D_{B\to A}p_{A}D_{A\to o},$$ (4)

From this, the reflection coefficient $r=\frac{M_{21}}{M_{11}}$ can be derived, and the reflectivity $R={\left|r\right|}^{2}$ is then calculated. Although the absorption effects of the sensing layer may affect sensitivity, their quantitative impact on sensing performance is strategically excluded from the theoretical model to simplify the analysis, with the focus placed on investigating the core sensing mechanisms. Although the transfer matrix method (TMM) employed in this study can conduct rigorous and efficient analysis of the overall structure’s reflectivity, it cannot directly address the local electromagnetic field distribution problem at the BDS-electrolyte interface. A comprehensive full-wave simulation (e.g., FEM) is slated as the immediate next step in our research plan to quantitatively map the surface plasmon polariton (SPP) profile at the BDS interface and provide a complete visualization of the near-field enhancement. This work will build directly upon the optimized design parameters identified in this theoretical study.

Sensitivity, as a key performance metric in biosensing systems, not only governs the ability to distinguish subtle changes in target analytes but also essentially guides device optimization for practical detection scenarios. For the proposed structure, sensitivity is operationally defined as:

$$S=\frac{\Delta \theta }{\Delta n_{S}},$$ (5)

where Δθ denotes the resonant angle shift and $\Delta n_{S}$ represents the variation in the refractive index of HSA. To isolate the core sensing mechanism-the shift of the Fano resonance due to changes in the real part of the refractive index-the model strategically neglects the absorption effects and dielectric dispersion of the sensing layer. This simplification is common in foundational theoretical studies of photonic biosensors as it allows for a clearer analysis of the fundamental relationships between structure, material properties (like the BDS Fermi level), and sensitivity. The impact of these omissions on the quantitative accuracy of the spectrum is acknowledged and will be addressed in future experimental work through calibration.

## 3 Results

**FIGURE 2:**
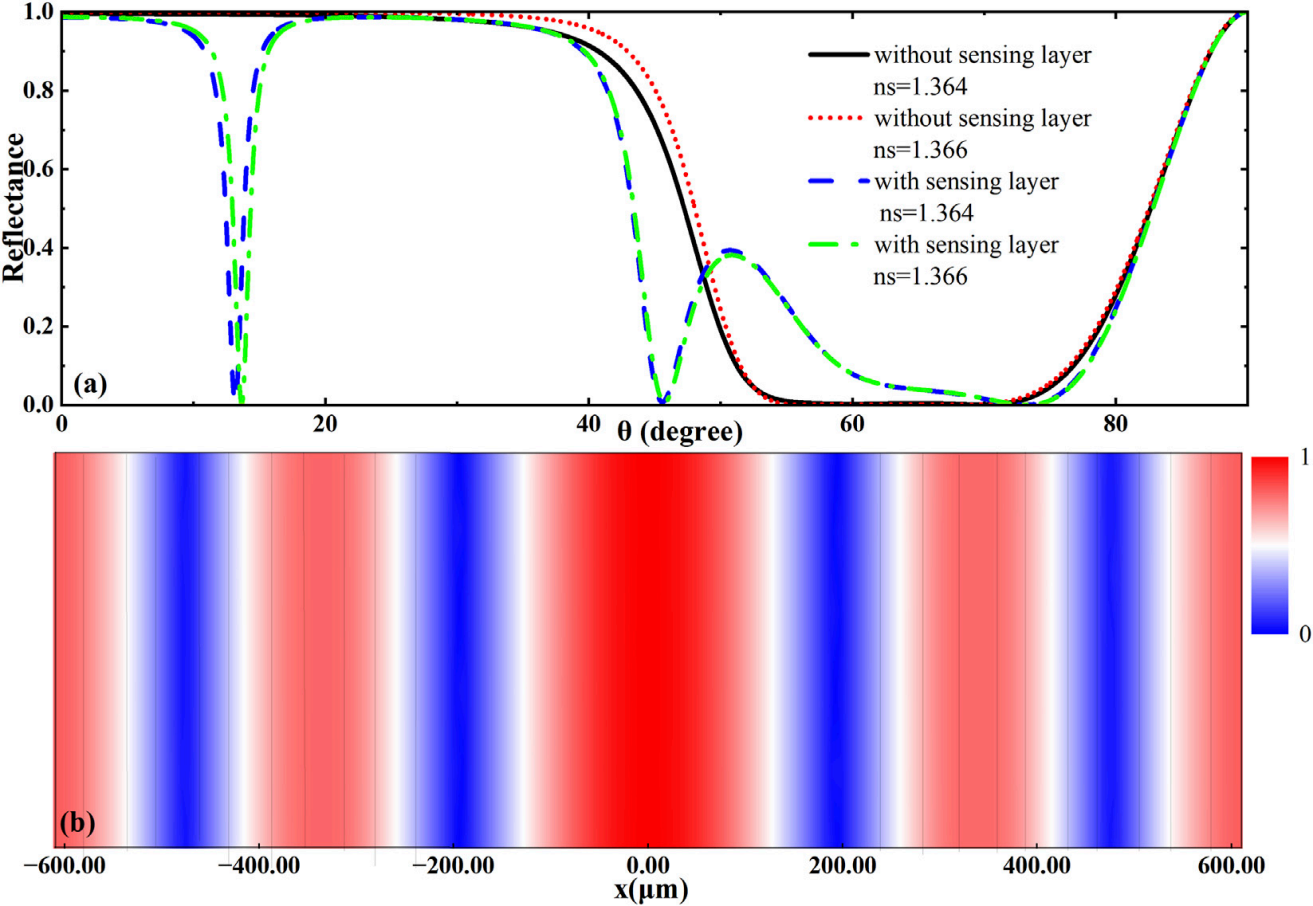
**(a)** The Reflectance spectra of the heterostructure; **(b)** Field strength distribution of the heterostructure.

The initial structure without the sensing layer and Dirac semimetal exhibited no distinct reflection dips across the investigated frequency range, as shown in Figure 2a. Upon integration of the sensing layer, pronounced reflection dip emerged, which exhibited significant shifts in response to variations in the refractive index of the analyte. The incorporation of the BDS not only enhanced the interaction between the incident terahertz wave and the biomolecular layer, but also provided an effective means for dynamically tuning the sensitivity of the biosensor via external modulation. To further investigate the resonance mechanism of the biosensor structure, the normalized electric field distribution at the central wavelength was simulated based on the parameters in Figure 1. The results are presented in Figure 2b, which shows the field profile across the structure using a line plot. For clarity in interpreting the field distribution in different media, the position of the sensing layer was set as the reference point (x = 0). In Figure 2b, different material regions are shaded with distinct background colors according to their spatial positions. A pronounced electric field enhancement is observed within the sensing layer, confirming the formation of a defect mode with characteristic field localization. The normalized electric field (E/E_0_) reaches a pronounced maximum (approximately 2.5 × 10^−5^) within the sensing layer, demonstrating strong field confinement and identifying it as the defect mode’s localization region. Additionally, field enhancement is also present near the BDS layer, indicating its role in enhancing light-matter interaction. These field distribution features effectively validate the reflection spectrum shown in Figure 2a and suggest synergistic effects between the defect mode and the plasmonic response, which contribute to the high sensitivity of the biosensing platform. It is important to note that our theoretical model employs a non-dispersive, lossless dielectric function for the HSA layer to highlight the fundamental sensing principle. In a practical device, the absorption and gentle dielectric dispersion of the protein-water solution in the THz regime would quantitatively affect the depth and width of the resonance dip. However, since our sensing signal is defined primarily by the resonance angle shift, which is dominantly governed by the real part of the refractive index, the key conclusions regarding sensitivity and tunability remain valid. Future work focused on direct experimental comparison will incorporate a comprehensive dispersive complex dielectric model to achieve precise spectral fitting.

**FIGURE 3:**
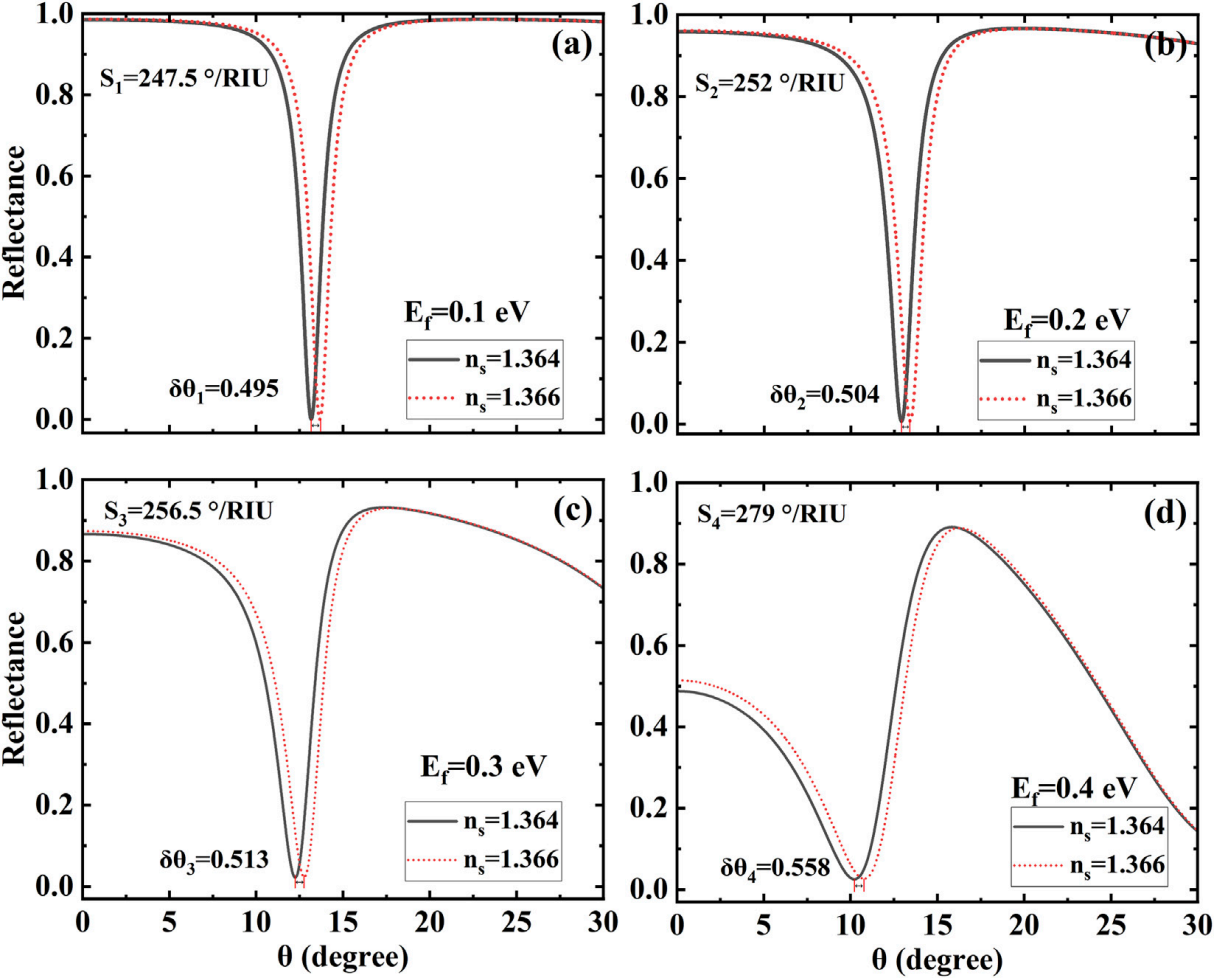
Reflectance spectra at different Fermi levels **(a)** 0.1 eV, **(b)** 0.2 eV, **(c)** 0.3 eV, **(d)** 0.4 eV. All parameters are consistent with those in Figure 2.

Subsequently, the sensitivity characteristics of the overall sensor structure are examined. For the sensing layer functionalized with biomolecules, the change in refractive index originates from intermolecular interactions, and the magnitude of this change is smaller compared to the values adopted in our previous studies. Figure 3 presents the reflection spectra corresponding to Fermi levels of 0.1 eV, 0.2 eV, 0.3 eV, and 0.4 eV, which form the basis for sensitivity calculation. As observed in Figure 3, sharp Fano resonance peaks emerge at specific refractive index values of the sensing layer under various Fermi levels. These sharp resonances indicate that even minor variations in structural parameters can induce significant shifts in the resonance angle. It is observed that subtle changes in biochemical properties leading to variations in the refractive index of the sensing layer result in a pronounced angular shift of the Fano resonance peak toward larger angles. The shift exceeds a certain threshold, and the sensitivity derived from this process reaches over 247.5°/RIU. This suggests a strong dependence of the mode-coupled Fano resonance on structural and material parameters, which is particularly advantageous for high-sensitivity biosensing applications. The Fermi level can be tuned by applying an external voltage, providing a convenient means for dynamic sensitivity adjustment. Notably, the device sensitivity increases with the Fermi level. However, beyond a certain value, distortion occurs in the reflection spectrum, which may compromise the reliability of sensitivity characterization under such conditions. Specifically, a closer inspection of the reflection dips in Figures 3c,d reveals a discernible asymmetry, which characterized a typical Fano resonances. A detailed quantitative analysis of the Fano asymmetry parameter q as a function of the Fermi level, which will provide further profound insight into the coupling strength, constitutes a key objective for our subsequent research, which will combine advanced fitting algorithms with full-wave electromagnetic simulations.

**FIGURE 4:**
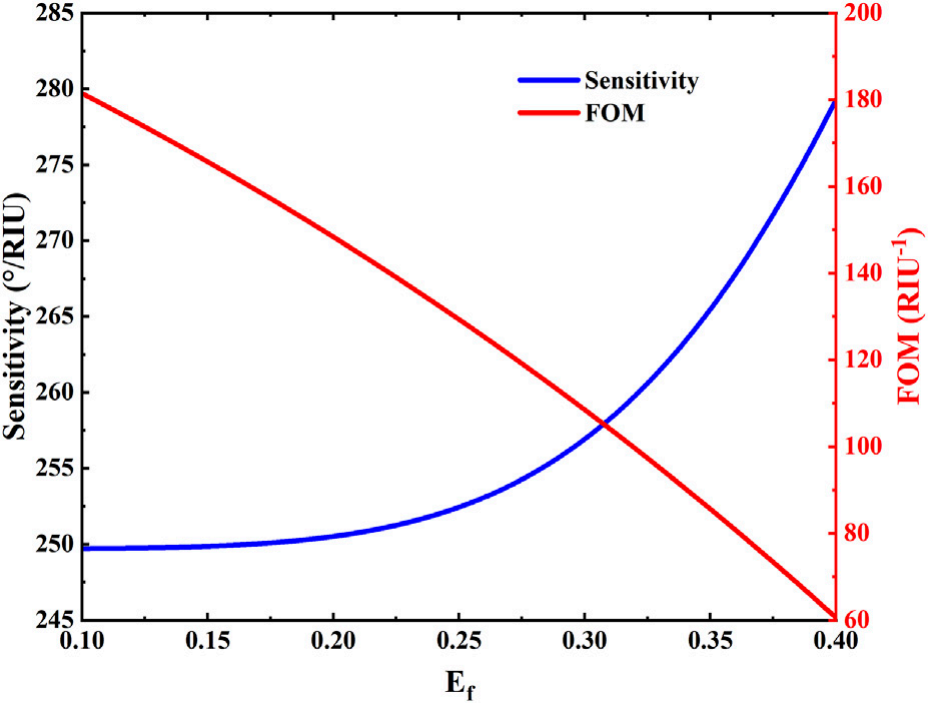
The sensitivity and FOM (Figure of Merit) of biosensor structure relative to the Fermi energy.

Figure 4 illustrates the variation of the sensitivity and the figure of merit (FOM) of the biosensor with respect to the Fermi level of the Dirac semimetal. Within the Fermi level range of 0.1 eV–0.4 eV, both parameters exhibit distinct variation trends. As the Fermi level increases, the sensitivity shows significant enhancement, which can be attributed to modifications in the dielectric properties and carrier concentration of the material, leading to a stronger plasmonic resonance effect. Even at a Fermi level as low as 0.1 eV, the structure maintains high sensitivity. However, the FOM gradually decreases with rising Fermi level, primarily due to increased optical loss and resonance broadening. The FOM serves as a comprehensive performance metric that combines both sensitivity and resonance linewidth—where high sensitivity and narrow full width at half maximum (FWHM) are desirable. This trend reflects a trade-off between high response intensity and overall sensing quality. Furthermore, as supported by the reflection spectra in Figure 2, neither sensitivity nor FOM increase indefinitely with the Fermi level. Practical limitations in doping and fabrication restrict the Fermi level to a relatively low range, while excessively high Fermi levels may induce mode mismatch, weakened coupling, and degradation of resonance conditions. Thus, selecting an appropriate Fermi level that balances sensitivity and FOM is essential for optimizing the sensor performance in the design stage. Thus, selecting an appropriate Fermi level that balances sensitivity and FOM is essential for optimizing the sensor performance in the design stage. This observed trade-off stems from the fundamental competition between plasmonic resonance enhancement and increased optical loss at higher Fermi levels, which broadens the resonance linewidth. While intrinsic to electro-optically active materials like BDS, this compromise is not a failure but a design parameter, enabling dynamic prioritization between ultimate sensitivity and optimal measurement precision in practical applications.

**FIGURE 5:**
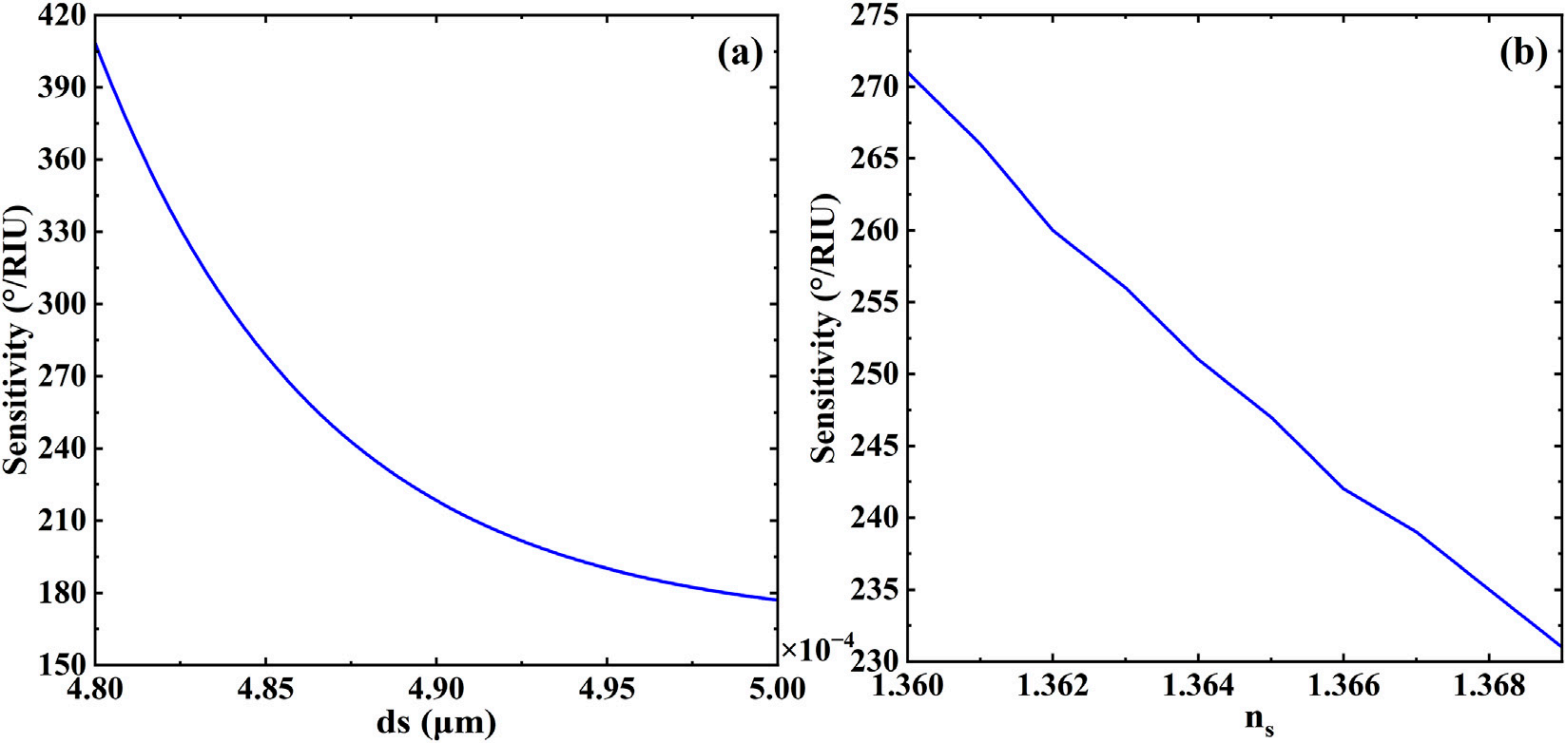
Effects of **(a)** the thickness and **(b)** the refractive index of the sensing layer on the sensitivity of biosensor. Other parameters are the same as in Figure 2.

Figure 5 illustrates the variation of sensitivity with respect to both the thickness (d_s_) and the refractive index (n_s_) of the sensing layer. As shown in Figure 5a, the sensitivity of the biosensor exhibits a clearly monotonic decreasing trend as the thickness of the sensing layer (d_s_) increases. This behavior suggests that reducing d_s_ is an effective strategy for achieving higher sensitivity. However, this does not imply that an excessively small thickness is optimal. On the one hand, extremely thin sensing layers impose stricter requirements on fabrication precision and process control. On the other hand, since d_s_ is closely linked to the spectral position of the defect mode, a significant reduction in thickness may lead to the gradual disappearance of the Fano resonance peak, thereby weakening the sensing capabilities of the structure. Hence, the values of d_s_ considered in our study are maintained above a certain threshold to ensure structural and functional integrity. Furthermore, as depicted in Figure 5b, an increase in the refractive index of the sensing layer (n_s_) also results in a gradual decrease in sensitivity. Specifically, when the refractive index increases from 1.36 to 1.369, the sensitivity shows a declining trend with slight fluctuations. This non-sharp decrease may be attributed to the complex interplay between biomolecular interactions and the evolving optical field distribution within the defect mode region. Such fluctuations could also originate from minor variations in the coupling strength between the defect mode and the external continuum under different dielectric conditions. These observations underscore the importance of carefully optimizing both physical and optical properties of the sensing layer to achieve consistent and high sensor performance.

## 4 Discussion

This study presents a systematic investigation of a BDS-integrated terahertz biosensor for label-free detection of human serum albumin (HSA). The sensor architecture combines a photonic crystal structure with BDS, achieving high sensitivity enabled by a Fano resonance resulting from the synergistic coupling between a defect mode and BDS-mediated plasmonic response. Electric field simulations verify significant field confinement in the sensing layer. The electrically tunable properties of BDS allow dynamic reconfiguration of sensor performance. By adjusting the Fermi level of BDS between 0.1 eV and 0.4 eV, the sensitivity can be enhanced, albeit at the cost of a reduced figure of merit (FOM) due to broader resonance peaks and increased loss, necessitating a balanced approach for practical applications. Structural optimization reveals that the sensitivity is inversely proportional to the sensing layer thickness (d_s_) and gradually decreases with increasing refractive index (n_s_). Beyond highly sensitive biosensing, the physical mechanisms underpinning our platform-specifically, the strong electric field confinement within the sensing region (Figure 2b)-suggest promising potential for advanced theranostic applications, particularly with nanozymes. This prospect is grounded in well-established physico-chemical principles: first, the local electric field intensity is a key factor in plasmon-enhanced catalysis, where it can significantly accelerate reaction rates by facilitating electron transfer or generating hot carriers. The field enhancement we demonstrate provides a theoretical basis for similarly boosting the catalytic efficiency of nanozymes positioned within the sensing volume. Second, the intensity of Raman scattering, a powerful technique for molecular imaging, scales non-linearly with the local field (approximately with the fourth power, |E|^4^). Our platform could, therefore, potentially enable dramatically enhanced imaging sensitivity for nanozyme-based contrast agents via a surface-enhanced Raman scattering (SERS) mechanism. While the experimental validation of these theranostic functions constitutes a critical direction for our future work, the current theoretical study solidly outlines a versatile and tunable platform upon which such multifunctional capabilities can be built.

## Data Availability

The original contributions presented in the study are included in the article/supplementary material, further inquiries can be directed to the corresponding authors.
